# Analysis of Clinical Characteristics of 556 Spinal Tuberculosis Patients in Two Tertiary Teaching Hospitals in Guangxi Province

**DOI:** 10.1155/2021/1344496

**Published:** 2021-12-10

**Authors:** Hao Zeng, Yingfang Liang, Jiaguo He, Liyi Chen, Haixia Su, Simi Liao, Sichun Huang, Hongyu Qin

**Affiliations:** ^1^Department of Spine and Osteopathy Surgery, Guangxi Medical University First Affiliated Hospital, 6#Shuangyong Rd, Nanning, 530000 Guangxi, China; ^2^Guangxi Key Laboratory of Regenerative Medicine, Guangxi Medical University, 22#Shuangyong Rd, Nanning, 530000 Guangxi, China; ^3^Department of Comprehensive Internal Medicine, Guangxi Medical University Affiliated Tumor Hospital, 71#Hedi Rd, Nanning, 530000 Guangxi, China; ^4^Department of Rehabilitation Medicine, Guangxi Medical University First Affiliated Hospital, 6#Shuangyong Rd, Nanning, 530000 Guangxi, China; ^5^Department of Statistics, School of Public Health, Guangxi Medical University, 22#Shuangyong Rd, Nanning, 530000 Guangxi, China; ^6^Department of Nutrition and Food Hygiene, School of Public Health, Guangxi Medical University, 22#Shuangyong Rd, Nanning, 530000 Guangxi, China

## Abstract

Spinal tuberculosis (STB), which accounts for half of musculoskeletal tuberculosis, is among the leading causes of extrapulmonary tuberculosis. Guangxi Province, located in southern China, is among the most severely affected provinces in China. In this study, we collected and analyzed data from 2 Class-A tertiary teaching hospitals in Nanning City, Guangxi Province, from 2011 to 2019, with the aim of providing reference points for the prevention, diagnosis, treatment, and prognosis analysis of STB, using the epidemiological characteristics of 556 STB cases. Our results revealed that males had a slightly higher incidence (50.17%) compared to females (49.83%), with 64.93% of cases falling between the ages of 18 and 45 years. Cases from rural communities accounted for 63.49% of the reviewed cases. The average time between onset of symptoms and hospitalization was 18.0 months (range: 1 day-220 months). The most commonly reported symptoms were lower back pain (78.60%), radicular pain (51.98%), and systemic toxemia (43.53%). Additionally, 53.98% of the reviewed cases had varying degrees of neurological impairment. The main pathological lesion locations were the lumbar spine (43.53%) and thoracic spine (32.55%). Among them, 72.66% of cases involved at least 2 vertebral segments, and 62.77% of cases presented with paravertebral abscesses. Among the cases reviewed, 90.65% underwent antituberculosis chemotherapy prior to surgery. Following treatment, the cure rate was 78.41%, while 3.78% of patients had postoperative relapse. There were cases of concomitant illnesses among the cases reviewed, 40.65% of patients also had pulmonary tuberculosis, 15.29% had hepatitis B, 13.30% had diabetes, and 7.91% had hypertension. Our results still demonstrate that spinal tuberculosis remains a serious public health problem in Guangxi Province. Thus, preventive measures should be directed towards rural residents with comorbidities such as the elderly and diabetic.

## 1. Introduction

Tuberculosis (TB) continues to be an infectious disease nightmare in developing countries. Late treatment of pulmonary tuberculosis still continues to pose a serious threat to public health and socioeconomic development in the developing world as it leads to incapacitation and uncontrolled spread [1]. Spinal tuberculosis (STB), which accounts for approximately 50% of musculoskeletal tuberculosis, is the most common extrapulmonary tuberculosis [2]. The diagnosis of spinal tuberculosis is relatively difficult. Pathological evidence from tissue samples is the gold standard for diagnosis, but in fact, it is very difficult to identify Mycobacterium tuberculosis from the lesion [2].

In addition, because of the vague nature of the presenting symptoms and paucity of neurological symptoms and signs in the early stages of the disease, most patients report serious manifestations like irreversible neurological impairments such as paralysis at the time of reporting, which increases the length of stay and treatment costs, resulting in serious financial burden [3].

Antituberculous chemotherapy remains the fundamental treatment modality for STB, with surgery as an adjuvant [4, 5]. According to the World Health Organization report, although the incidence and mortality rates of tuberculosis (TB) in China have been declining steadily in recent years, China still remains one of the countries with the highest TB burden in the world [6]. There were approximately 833,000 new tuberculosis cases in China in 2019, with a reported 388,000 mortalities, demonstrating that tuberculosis is still a serious public health problem among Chinese people [6].

The epidemiology of tuberculosis is influenced by factors such as geographic location, lifestyle, socioeconomic development, and the availability of good medical services. Guangxi Province, located in southern China, has a population of over 50.12 million, with a rural population of 33.22 million according to 2021 population data. Guangxi Province is among the provinces with the highest tuberculosis burden in China, with a 2019 annual incidence of 81.12/100,000 compared to the national incidence of 44.45/100,000. This therefore necessitates more studies into the prevention and treatment measures of tuberculosis-related diseases in the province. There are currently few epidemiological investigations into spinal tuberculosis in Guangxi; thus, this study investigates this common extrapulmonary disease in order to better understand the incidence, as well as provide references for prevention and treatment in the province and beyond. This study collected and analyzed inpatient data such as clinical symptoms, diagnosis, treatment, and prognosis of 556 confirmed spinal TB cases from 2 tertiary teaching hospitals in Nanning City, in order to investigate the stated goals.

## 2. Materials and Methods

### 2.1. Patient Data

We retrospectively analyzed the medical records of STB patients admitted to 2 tertiary teaching hospitals from January 2011 to December 2019 using the medical record retrieval code. The collected data included clinical features such as pain, radiculopathy, systemic symptoms, neurological manifestations, imaging features, pathologic vertebrae, treatment options, and posttreatment complications. Other clinical data such as comorbidities, as well as prognostic indicators, were also analyzed. Personal information such as gender, age, residence (rural or urban), and occupation was also analyzed. The Frankel grading system was used to assess the grades of the neurological manifestations in the reviewed patients; grade A: complete neurological deficit; grade B: sparing of sensation; grade C: sparing of sensation but no useful motor function; grade D: sparing of sensation and useful motor function; and grade E: no deficits. Since pulmonary tuberculosis (PTB) and diabetes are common comorbidities of STB, we analyzed the correlation between PTB, diabetes, and STB. Guangxi Province also has a high incidence of hepatitis B and hypertension; hence, their correlations were also analyzed.

### 2.2. Diagnosis and Treatment

The diagnostic criteria for STB are as follows: (1) clinical signs and symptoms, such as back pain, spinal deformity, neurological deficits (numbness, weakness, hypoesthesia, dyskinesia, and paralysis), and systemic toxemic symptoms (low-grade fever, night sweats), and weight loss; (2) CT and MRI findings: vertebral destruction, intervertebral space involvement, kyphosis, paravertebral abscess, etc.; (3) laboratory findings: anemia, elevated erythrocyte sedimentation rate (ESR) and C-reactive protein (CRP), positive T-SPOT, tuberculosis antibodies (IgG and IgM), and positive tuberculin skin test; (4) diagnostic antituberculosis chemotherapy which was effective in difficult to diagnose cases; and (5) pathological evidence from tissue samples which is the gold standard for diagnosis.

All patients received the combination of the 4 antituberculosis chemotherapeutic agents HREZ (rifampicin (450 mg/day), isoniazid (300 mg/day), ethambutol (750 mg/day), and pyrazinamide (750 mg/day)) for at least 18 months. All patients underwent rigorous monitoring of renal and liver functions during treatment. All patients and their immediate families were also educated and trained to understand the importance of early, regular, and full completion of the combined medication, and family members were urged to help patients adhere to their medications. Patients were also advised to rest and avoid strenuous activities. None of the patients included in the study were HIV positive.

Patients were evaluated by telephone follow-up and outpatient visitation to gather information on posttreatment prognosis. Patients were classified as “cured” if there is no recurrent TB lesion within 2 years after treatment, the ESR level remained within the normal range, bone healing is observed on follow-up imaging examinations, and the clinical symptoms have disappeared for at least 3 months [7]. Relapses were presented as postoperative recurrence of TB lesions, sinus formation, progressive kyphosis, abscess formation, or internal fixation fractures. Patients were classified as “other” because of incomplete follow-up data, loss of contact, failure to heal, transferred treatment, or death [8].

### 2.3. Statistical Analysis

Demographic and clinical characteristics were collected and included gender, age, place of residence, degree of education, neurological deficit, pain, radiculopathy, systemic symptoms, neurological manifestations, imaging features, pathologic vertebrae, treatment options, posttreatment complications, Hb, and ESR. All statistical analyses were performed using SPSS statistical software version 26.0 (IBM, Chicago, USA). The chi-squared (*χ*^2^) test was used to analyze the collected data, with *p* < 0.05 considered statistically significant.

## 3. Results

### 3.1. General Information

A total of 556 eligible cases of confirmed STB were reviewed in this study (Table 1). Among them, there were 361 (64.93%) patients between the ages of 18 and 45 years and 98 (17.63%) patients between 46 and 60 years. There was a statistically significant difference between the age distribution of patients (*p* = 0.003) during the reviewed period. During this 9-year period, the incidence of STB was relatively stable (30-112 new cases per year), and the gender proportions, male (50.17%) and females (49.83%), remained virtually unchanged, without any statistically significant differences (*p* > 0.05). Among the cases reviewed, rural residents accounted for 63.49%, while urban residents accounted for 36.51%, but the difference was not statistically significant (*p* > 0.05). In terms of occupational distribution, there were 338 farmers (60.79%), 89 blue-collar workers (16.01%), 26 students (4.68%), and 103 (18.53%) in other forms of occupation. The result of our correlation analysis between occupation and years was statistically significant (*p* = 0.036). The average interval between onset of symptoms and hospitalization was 18.0 months (range: 1 day-220 months). The average intervals for rural patients were significantly longer (25.5 months) compared to urban patients (9.1 months) (*p* < 0.05).

**Figure 1:**
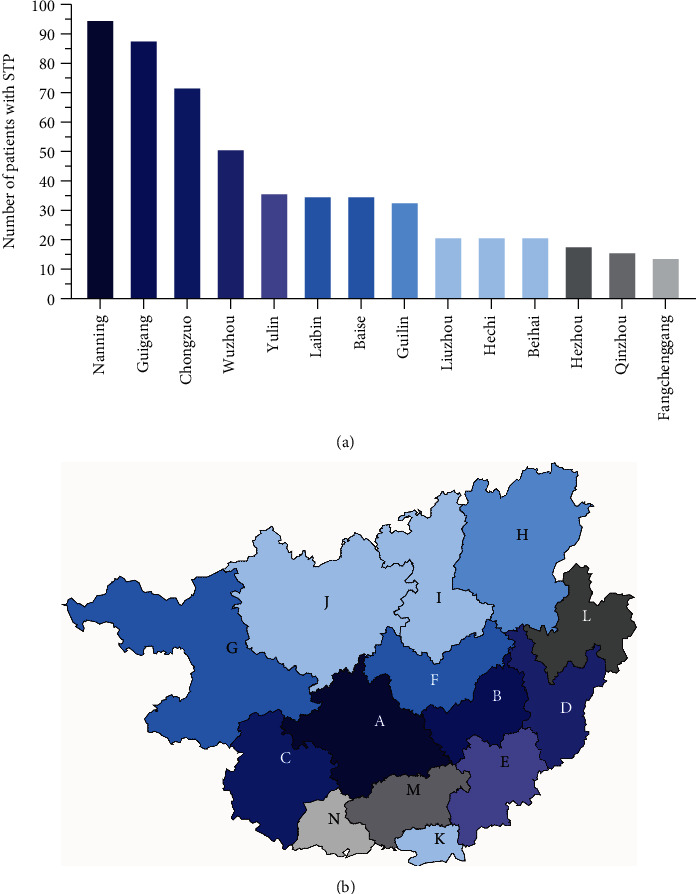
The geographical location and the number of patients in the main cities of Guangxi. (a) The number of STB patients in major cities. Nanning 17.09%, Guigang 15.83%, Chongzuo 12.95%, Wuzhou 9.17%, Yulin 6.47%, Laibin 6.29%, Baise 6.29%, Guilin 5.94%, Liuzhou 3.78%, Hechi 3.78%, Beihai 3.78%, Hezhou 3.24%, Qinzhou 2.88%, and Fangchenggang 2.51%. (b) The geographical location of major cities: Nanning (A), Guigang (B), Chongzuo (C), Wuzhou (D), Yulin (E), Laibin (F), Baise (G), Guilin (H), Liuzhou (I), Hechi (J), Beihai (K), Hezhou (L), Qinzhou (M), and Fangchenggang (N).

There is regional and local distribution of patients in the major cities of Guangxi Province. During the period studied, Nanning City had the highest incidence of STB (17.09%), followed by Guigang 15.83%, Chongzuo 12.95%, Wuzhou 9.17%, Yulin 6.47%, Laibin 6.29%, Baise 6.29%, Guilin 5.94%, etc. Figures 1(a) and 1(b).

### 3.2. Clinical Data Analysis

The clinical manifestations of the STB patients involved in this study are summarized in Table 2. Lower back pain was reported in 78.60% of patients, followed by radicular pain (51.98%) and systemic toxemic symptoms (43.53%). The results of our correlation analysis revealed no correlation between lower back pain, systemic poisoning, and annual incidence (years) during the period of the study (*p* > 0.05). However, there was a statistically significant correlation between radicular pain and annual TB incidence (*p* < 0.05).

According to the Frankel classification, there were 15 (2.7%) grade A, 25 (4.5%) grade B, 56 (10.07%) grade C, 204 (36.69%) grade D, and 256 grade E (46.04%) cases (Table 2). However, there was no statistically significant difference between the Frankel grades and annual disease incidence (*p* > 0.05). During follow-up examination, 379 patients (68.17%) of the 556 reviewed cases reported vertebral tenderness during physical examination; also, 367 patients (66.01%) reported vertebral percussion pain. In 153 patients (27.52%), kyphosis deformities were observed during physical examination.

### 3.3. Radiographic and Laboratory Examinations

The summary of the analysis of the radiographic findings is shown in Table 3. The most common pathologic lesion locations were lumbar spine (43.53%), thoracic spine (32.55%), thoracolumbar spine (6.65%), cervicothoracic spine (6.29%), sacral spine (4.14%), lumbosacral spine (3.96%), and cervical spine (2.88%). There was no statistically significant difference in pathological location and annual incidence (*p* > 0.05). Among the cases reviewed, 72.66% involved 2 vertebral segments, while 16.73% involved 3 or more segments. Abscesses were detected using CT/MRI, with 349 of the 556 patients (62.77%) diagnosed with paravertebral, prevertebral, or psoas major abscesses.

The normal range of hemoglobin is 115-150 g/L. In this study, patients' hemoglobin levels ranged between 61 and 164 g/L. Among the cases reviewed, 50.76% had Hb ranging between 90 and 115 g/L; 3.12% had Hb ranging between 80 and 90 g/L, while 1.03% were below 80 g/L. Patient ESR ranged between 2 and 152 mm/h. In 109 patients, it was below 20 mm/h, while it ranged between 20 and 100 in 301 patients, with 146 patients having ESR higher than 100 mm/h. The range for CRP in this study was 0.11-204.31 mg/L. Among the cases reviewed, there were 127 patients (22.84%) with normal CRP, while the rest had varying degrees of CRP elevation, with the highest being 204.31 mg/L.

### 3.4. Treatment and Prognosis

**Figure 2:**
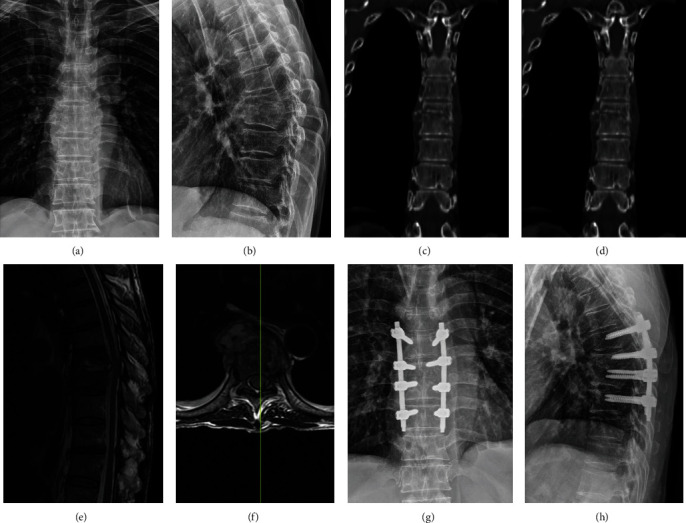
A 68-year-old male with T5/6 lesions. (a–f) The preoperative imaging data showed T5/6 vertebral bodies' destructions with kyphosis deformity and spinal cord severely compressed. The postoperative sagittal and coronal X-ray (g, h) indicated that the kyphosis got obviously improved.

The treatment options administered are summarized in Table 4. In 504 (90.65%) of patients, preoperative antituberculosis chemotherapy was used prior to surgical treatment and achieved good efficacy (Figure 2), while 52 (9.35%) patients were treated with only antituberculosis chemotherapy. During the 9-year period, patients who underwent chemotherapy and surgery were greater than those treated with only chemotherapy; however, there was no statistically significant difference between them (*p* > 0.05). The primary surgical methods were anterior debridement, bone grafting, and internal fixation; anterior-lateral debridement, bone grafting, and internal fixation; and posterior debridement, bone grafting, and internal fixation.

The summary of patients' prognoses is shown in Table 4. A total of 486 patients (78.41%) were classified as “cured” after receiving treatment. Local recurrence was observed in 21 patients (3.78%). Such patients received continued antituberculosis chemotherapy and underwent revision surgery. 49 (8.81%) patients were classified as “other” because of incomplete follow-up data, loss of contact, failure to heal, or transferred treatment. No patient died during the entire follow-up period.

### 3.5. Comorbidities

**Figure 3:**
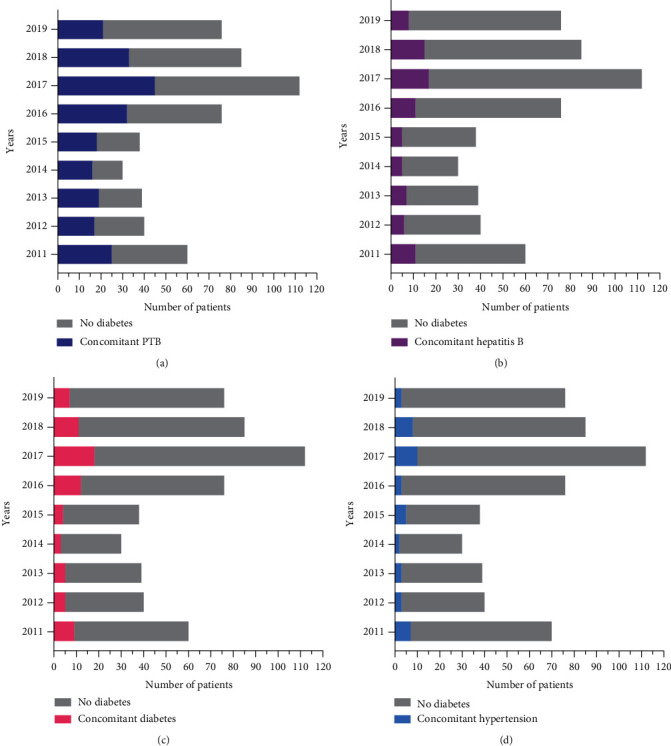
Comorbidities. (a) The number of patients with concomitant PTB. (b) The number of patients with concomitant hepatitis B. (c) The number of patients with concomitant diabetes. (d) The number of patients with concomitant hypertension.

Among the 556 patients reviewed, 226 (40.65%) had PTB, 85 (15.29%) had hepatitis B, 74 (13.30%) had diabetes, and 44 (7.91%) had hypertension (Figures 3(a)–3(d)).

Among the patients with pulmonary tuberculosis (Table 5), 64.60% were males and 35.4% were females. Moreover, 40.71% of them reported symptoms of systemic toxemia, 69.91% had paravertebral abscesses, and 75.66% had neurological impairments. Surgical treatment was performed in 86.73% of the pulmonary tuberculosis patients, of which 82.30% were classified as “cured” after the treatment. The results of our correlation analysis revealed that there were statistically significant differences between gender, paravertebral abscess, nerve injury, surgery, and cure rate in patients with pulmonary TB diagnosis (*p* < 0.05). On the contrary, there was no statistically significant correlation between symptoms of systemic toxemia and pulmonary tuberculosis diagnosis (*p* > 0.05).

Among the patients reviewed, 48.64% had diabetes (Table 6). Among which, patients 45 years old and over accounted for 82.43%, with 43.24% of them reporting systemic toxemic symptoms. Moreover, neurological manifestations were reported in 52.70%, while paravertebral abscesses were observed in 71.62%. Following surgical treatment, 78.37% of the diabetic patients were classified as “cured.” Age, surgical treatment, and cure rate were found to be correlated with diabetes (*p* < 0.05). However, systemic toxemia, paravertebral abscesses, and neurological impairments were not significantly correlated with diabetes (*p* > 0.05).

## 4. Discussion

Tuberculosis continues to threaten public health, especially in developing countries. Spinal tuberculosis (Pott disease) was first described in 1782 by Percival Pott [9]. In developing countries, spinal tuberculosis mostly occurs among young and middle-aged people, while in the developed world, the elderly are the most affected [10, 11]. An epidemiological survey of spinal tuberculosis in the United States between 2002 and 2011 revealed an average age of 51 years (35-65 years), with 24.5% of cases over the age of 65 years [12]. In this study, 64.93% of the patients were between the ages of 18 and 45 years, while 17.63% were between 46 and 60 years old. This is possible because patients in the age group of 18-60 years perform physical labor for a long time, which increases the load on the spine, which leads to chronic spinal injury, which can easily lead to infection of Mycobacterium tuberculosis. In this study, patients younger than 18 years accounted for only 7.01%, which may be explained by the fact that the protective effect of BCG vaccination continues into the adolescence stage [13]. According to a 2020 report by the World Health Organization [2], the male to female ratio of newly diagnosed tuberculosis in China was 2.19 : 1. However, in this study, the male to female ratio was 1 : 1, with no statistically significant change in the 9-year period of the study. In Guizhou, which is also an underdeveloped southwestern region of China, the ratio of male to female in STB is 1.1 : 1 [14]. This similarity in the sex ratio may be due to the similarities in the economic, medical, and educational levels of the two regions. In our study, the number of STB patients after 2016 has an increasing trend than before, but it is not statistically significant. This may be because in recent years, the country has strengthened poverty alleviation and the country has increased its medical insurance investment. The awareness of hospital visits has increased, so the number of visits has increased.

It is reported that 80% of STB patients worldwide are in poor and developing countries. Studies have also reported that 80% of STB patients in China are from rural areas [15]; however, in this study, the ratio was slightly lower at 63.48%. 60.79% of patients were farmers. The average interval between the onset of symptoms and hospitalization of rural patients was 25.5 months; on the contrary, that for the patient from urban areas was 9.1 months. This may be related to the low level of health awareness, education, and the overall level of socioeconomic development in Guangxi Province. According to the urban distribution of STB, Nanning City, Guigang City, and Chongzuo City have more STB patients than other major cities in China. These differences can be attributed to the lower standard of medical care and socioeconomic development in these cities.

Unlike pulmonary tuberculosis, the most common manifestation of spinal tuberculosis is lower back pain [16]. Other major symptoms include systemic toxemic symptoms such as low-grade fever, night sweats, and weight loss. In this study, 78.60% of patients reported lower back pain, while 43.53% reported systemic toxemic symptoms. The main reason why STB patients seek medical evaluation and treatment is lower back pain. In this study, vertebral tenderness was reported in 68.17%, while vertebral percussion pain was reported in 66.01% of patients during physical examination. Kyphosis was also observed in 27.52% during the physical. The contiguous bone necrosis associated with tuberculosis coupled with psoas, paravertebral, or prevertebral abscess formation may result in neurological manifestations such as back pain and radiating pain. Studies have reported that delayed treatment of STB could result in neurological damage and even paralysis [17]. In this study, 51.98% of patients reported neuropathic pain. Varying degrees of neurological impairment were observed in 53.96% of cases. Severe neurological impairments were observed in 7.2% of cases (2.70% Frankel grade A; 4.50% grade B). Following surgical treatment, varying degrees of neurological function recovery were observed in such patients, confirming the poorer recovery rate among patients with severe neurological impairment on admission.

According to the available literature, the most common site for spinal tuberculosis is the lumbar and thoracic spine [18]. In this study, lumbar tuberculous lesions accounted for 43.53%, while thoracic and thoracolumbar lesions accounted for 32.55% and 6.65%, respectively. This localization may be related to the anatomical factors of the high level of mobility and load forces in the lumbar region in the thoracic region. STB lesions usually involve two or more vertebrae. This is because the segmental arteries bifurcate to supply two adjacent vertebrae, making the hematogenous spread to adjacent vertebrae easy. MTB involvement of the anterior or posterior longitudinal ligament may also enable multiple adjacent vertebrae involvement [8]. In this study, 72.66% of cases involved 2 adjacent vertebrae, while 16.73% involved 3 or more vertebrae, while paravertebral abscesses were found in 62.77% of patients.

The hemoglobin level has been used as an indirect indicator of health and nutritional status, and studies have reported that tuberculosis causes weight loss and malnutrition [19]. Thus, in this study, we analyzed the hemoglobin levels to determine the nutritional status of patients. In this study, anemia was observed in 54.91% of patients, thus confirming the high incidence of malnutrition in tuberculosis patients. Thus, our results also revealed that more attention should be paid to nutritional supplementation before, during, and after antituberculosis chemotherapy or surgical treatment for TB-related diseases. ESR and CRP, which are used as indirect indicators of inflammation and inflammatory reactions, have high sensitivity but low specificity. However, they can still be used to monitor disease activity during follow-up. The average ESR is 43.5 mm/h [20]; however, in this study, ESR ranged between 2 and 152 mm/h. The average CRP in STB patients has been reported to range between 6 and 197 mg/L [21]. However, in this study, it ranged between 0.11 and 204.31 mg/L. Thus, it confirms that inflammatory reactions are common in tuberculosis patients and that ESR and CRP can be used as indirect indicators of postoperative prognosis.

Antituberculosis drugs are currently the basis for spinal tuberculosis treatment. Surgical treatment on the other hand plays an important adjuvant role. This is because surgical risks such as loss of life, infection, metastasis, and postoperative recurrence are significantly increased in the absence of preoperative chemotherapy [22]. The American Thoracic Society recommends 6-9 months of chemotherapy for STB, and many studies have demonstrated the effectiveness of such a program [23]. Surgical indications for STB include spinal cord and nerve root compression or injury, severe kyphotic deformity or progressive kyphosis, spinal column instability, and abscesses. Surgical decompression is recommended as soon as possible following effective chemotherapy; this not only promotes recovery of nerve function and spinal column stability but also provides specimens for confirmatory pathological examination of surgical samples to help guide chemotherapy. In this study, 90.65% of patients received preoperative antituberculosis, with a cure rate of 78.41%. The recurrence rate was 3.78% (21 patients). Reasons for relapse may include unstable antituberculosis drug treatment, malnutrition, comorbidities, and lack of rest. Relapsed patients were cured by revision surgery, including debridement, drainage, and local catheter infusion of antituberculosis drugs.

Studies have reported that between 33% and 50% of STB patients have a history of pulmonary tuberculosis [24, 25]. In this study, 40.65% of the STB patients reviewed had PTB history, the majority of them being males, who were also at risk of developing paravertebral abscess lesions and nerve damage. This could be attributed to the high incidence of smoking and drinking among Chinese men. Furthermore, the majority of patients reviewed in this study were farmers or had blue-collar jobs, where manual labor is the norm.

The immunosuppression observed in diabetes patients has been reported to result in increased susceptibility to infections and poor tissue repair abilities [26]. Active tuberculosis is an infection that can aggravate diabetes and may even induce acute ketoacidosis. Diabetes and tuberculosis patients usually have the characteristics of the long course, severe illness, rapid progress, slow recovery, etc., which are difficult to treat [27, 28]. In addition, patients with diabetes and STB have impaired anti-infection and tissue repair capabilities, which affect the prognosis and sometimes lead to surgical failure. In this study, the majority of STB patients over 45 years with diabetes were treated conservatively unless necessary.

This study has several limitations. Firstly, it is a retrospective study of 556 inpatients in 2 tertiary hospitals. Thus, the sample size is relatively small. Secondly, this study did not include outpatients, which may lead to a high proportion of surgically treated patients and no HIV/ARDS cases. Finally, this study did not obtain the results of antituberculosis drug resistance.

## 5. Conclusion

Spinal tuberculosis is still a serious problem that endangers public health. Lower back pain is the most common clinical manifestation of STB, and the lumbar vertebrae are the most commonly affected. More attention should be paid to rural residents, farmers, blue-collar workers, elderly, and diabetic patients who should be the main focus of spinal tuberculosis preventive measures, especially in underdeveloped areas. Our results provided valuable information on the epidemiological characteristics of spinal tuberculosis in Guangxi Province, China, thus helping to raise local, regional, and national attention to it.

## Figures and Tables

**Table 1:** Demographics and epidemiology of inpatients with STB.

Year	Age (years)				Gender		Residence		Occupation				Total
	<18	18~45	46~60	>60	Male	Female	Rural	Urban	Farmer	Worker	Student	Others	
2011	3	29	18	10	31	29	40	20	28	13	2	17	60
2012	3	25	8	4	21	19	26	14	22	5	1	12	40
2013	2	21	10	6	23	16	23	16	19	6	2	12	39
2014	2	17	7	4	17	13	17	13	15	4	1	10	30
2015	3	24	6	5	25	13	25	13	20	5	2	11	38
2016	7	51	11	7	31	45	45	31	55	10	5	6	76
2017	9	77	15	11	54	58	78	34	75	20	5	12	112
2018	6	59	12	8	36	49	50	35	54	12	6	13	85
2019	4	58	11	3	41	35	49	27	50	14	2	10	76
Total	39	361	98	58	279	277	353	203	338	89	26	103	556
Rate (%)	7.01	64.93	17.63	10.43	50.17	49.83	63.48	36.51	60.79	16.01	4.68	18.53	
*χ* ^2^	46.929				10.923		4.593		37.833				
*p*	0.003				0.206		0.800		0.036				

**Table 2:** Clinical manifestations of patients with STB.

Year	Clinical presentation						Neurological injury (Frankel classification)					Total
	Back pain		Radiating pain		Systemic toxicity symptoms							
	Yes	No	Yes	No	Yes	No	A	B	C	D	E	
2011	45	15	34	26	25	35	2	1	4	20	33	60
2012	31	9	26	14	19	21	1	3	5	13	18	40
2013	29	10	17	12	16	23	3	4	4	12	16	39
2014	19	11	15	15	17	13	1	2	5	9	13	30
2015	31	7	13	25	13	25	1	1	4	15	17	38
2016	63	13	54	22	30	46	1	2	6	22	45	76
2017	93	19	53	59	59	53	3	5	12	42	50	112
2018	65	20	42	43	38	47	1	3	7	39	35	85
2019	61	15	35	41	25	51	2	4	9	32	29	76
Total	437	119	289	257	242	314	15	25	56	204	256	556
Rate (%)	78.60	21.40	51.98	48.02	43.53	56.47	2.70	4.50	10.07	36.69	46.04	
*χ* ^2^	7.762		21.798		11.757		23.440					
*p*	0.457		0.005		0.162		0.864					

**Table 3:** Imaging findings of patients with STB.

Year	Lesion sites							Involved number of vertebrae			Abscess		Total
	C	CT	T	TL	L	LS	S	1	2	≥3	Yes	No	
2011	2	8	18	5	21	2	4	7	41	12	39	21	60
2012	2	4	10	4	15	2	3	6	25	9	23	17	40
2013	1	2	10	4	17	2	3	4	23	12	26	13	39
2014	1	1	8	3	14	2	1	5	21	4	21	9	30
2015	2	3	12	3	14	2	2	6	27	5	24	14	38
2016	1	2	27	4	37	2	3	7	57	12	48	28	76
2017	3	8	39	7	49	4	2	8	92	12	76	36	112
2018	2	3	30	4	42	2	2	9	65	11	49	36	85
2019	2	4	27	3	33	4	3	7	53	16	43	33	76
Total	16	35	181	37	242	22	23	59	404	93	349	207	556
Rate (%)	2.88	6.29	32.55	6.65	43.53	3.96	4.14	10.61	72.66	16.73	62.77	37.23	
*χ* ^2^	27.850							17.354			4.976		
*p*	0.991							0.363			0.760		

C: cervical; CT: cervicothoracic; T: thoracic; TL: thoracolumbar; L: lumbar; LS: lumbosacral; S: sacrococcygeal.

**Table 4:** Treatment and prognosis of patients with STB.

Year	Treatment		Prognosis			Total
	Only anti-TB chemotherapy	Anti-TB chemotherapy and surgery	Cured	Relapse	Others	
2011	6	54	56	3	1	60
2012	5	35	35	2	3	40
2013	7	32	34	3	2	39
2014	3	27	25	1	4	30
2015	4	34	37	0	1	38
2016	6	70	62	4	10	76
2017	11	101	98	3	11	112
2018	6	79	75	3	8	85
2019	4	72	64	2	9	76
Total	52	504	486	21	49	556
Rate (%)	9.35	90.65	78.41	3.78	8.81	
*χ* ^2^	5.835		17.847			
*p*	0.666		0.333			

**Table 5:** Correlation analysis between STB and PTB.

Variate	Type	With PTB		Without PTB		Total	*χ* ^2^	*p*
		*N*	%	*N*	%			
Gender	Male	146	64.60	133	40.30	279	31.680	<0.001
	Female	80	35.4	197	59.7	277		
Systemic toxicity symptoms	Yes	91	40.71	151	45.76	242	1.646	0.200
	No	135	59.29	179	54.24	314		
Abscess	Yes	158	69.91	131	39.70	349	49.059	<0.001
	No	68	30.09	199	60.3	207		
Neurological injury	Yes	171	75.66	129	39.09	300	72.219	<0.001
	No	55	24.34	201	60.91	256		
Surgery	Yes	196	86.73	308	93.33	504	6.908	0.009
	No	30	13.27	22	6.67	52		
Prognosis	Cure	186	82.30	300	90.90	486	9.032	0.003
	Other	40	17.7	30	9.1	70		

**Table 6:** Correlation analysis between STB and diabetes.

Variate	Type	With diabetes		Without diabetes		Total	*χ* ^2^	*p*
		*N*	%	*N*	%			
Age (years)	>45	61	82.43	95	19.71	156	125.032	<0.001
	<45	13	17.57	387	80.29	400		
Systemic toxicity symptoms	Yes	32	43.24	210	43.56	242	0.003	0.958
	No	42	56.76	272	56.44	314		
Abscess	Yes	53	71.62	296	61.41	349	2.862	0.091
	No	21	28.38	186	38.59	207		
Neurological injury	Yes	39	52.70	261	54.15	300	0.054	0.816
	No	35	47.3	221	45.85	256		
Surgery	Yes	36	48.64	468	97.1	504	177.603	<0.001
	No	38	51.36	14	2.90	52		
Prognosis	Cure	58	78.37	428	88.79	486	6.327	0.012
	Other	16	21.63	54	11.21	70		

## Data Availability

The data used to support the findings of this study are included within the article.
