# Corrigendum to “Analysis of Clinical Characteristics of 556 Spinal Tuberculosis Patients in Two Tertiary Teaching Hospitals in Guangxi Province”

**DOI:** 10.1155/bmri/9808104

**Published:** 2025-07-09

H. Zeng, Y. Liang, J. He, et al., “Analysis of Clinical Characteristics of 556 Spinal Tuberculosis Patients in Two Tertiary Teaching Hospitals in Guangxi Province,” *BioMed Research International*, no. 2021 (2021): 1344496, https://doi.org/10.1155/2021/1344496.

In the above article, published on 10 December 2021, there was an error in Figure 3. The keys to the figure panels a, b, and d have been updated as follows:

- Figure 3a key: updated from “No diabetes” to “No PTB”
- Figure 3b key: updated from “No diabetes” to “No Hepatitis B”
- Figure 3d key: updated from “No diabetes” to “No hypertension.”

The corrected figure is shown below:

We apologize for this error.

## Figures and Tables

**Figure 1:**
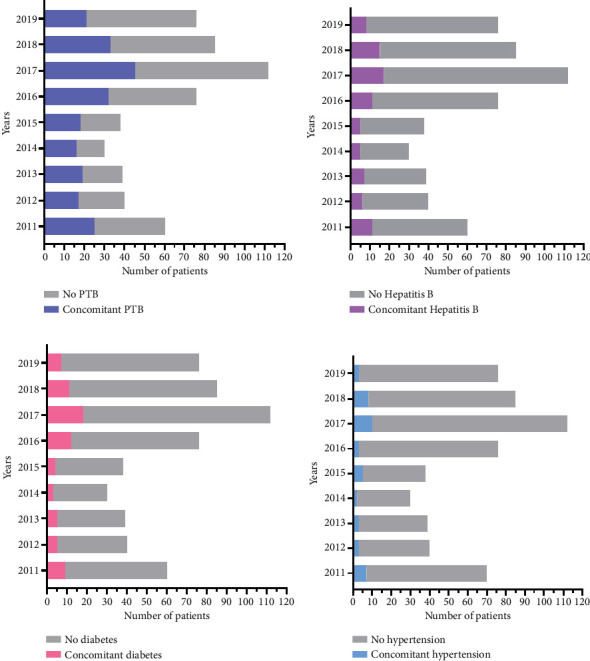
Comorbidities. (a) The number of patients with concomitant PTB. (b) The number of patients with concomitant Hepatitis B. (c) The number of patients with concomitant diabetes. (d) The number of patients with concomitant hypertension.

